# Constraints on superoxide mediated formation of manganese oxides

**DOI:** 10.3389/fmicb.2013.00262

**Published:** 2013-09-03

**Authors:** Deric R. Learman, Bettina M. Voelker, Andrew S. Madden, Colleen M. Hansel

**Affiliations:** ^1^Institute for Great Lakes Research and Department of Earth and Atmospheric Sciences, Central Michigan UniversityMount Pleasant, MI, USA; ^2^Department of Chemistry and Geochemistry, Colorado School of MinesGolden, CO, USA; ^3^School of Geology and Geophysics, University of Oklahoma, NormanOK, USA; ^4^Marine Chemistry and Geochemistry Department, Woods Hole Oceanographic InstitutionWoods Hole, MA, USA

**Keywords:** manganese oxidation, manganese oxides, superoxide, reactive oxygen species, Mn(III) complexes, organic ligands

## Abstract

Manganese (Mn) oxides are among the most reactive sorbents and oxidants within the environment, where they play a central role in the cycling of nutrients, metals, and carbon. Recent discoveries have identified superoxide ($O_{2}^{-}$) both of biogenic and abiogenic origin as an effective oxidant of Mn(II) leading to the formation of Mn oxides. Here we examined the conditions under which abiotically produced superoxide led to oxidative precipitation of Mn and the solid-phases produced. Oxidized Mn, as both aqueous Mn(III) and Mn(III/IV) oxides, was only observed in the presence of active catalase, indicating that hydrogen peroxide (H_2_O_2_), a product of the reaction of $O_{2}^{-}$ with Mn(II), inhibits the oxidation process presumably through the reduction of Mn(III). Citrate and pyrophosphate increased the yield of oxidized Mn but decreased the amount of Mn oxide produced via formation of Mn(III)-ligand complexes. While complexing ligands played a role in stabilizing Mn(III), they did not eliminate the inhibition of net Mn(III) formation by H_2_O_2_. The Mn oxides precipitated were highly disordered colloidal hexagonal birnessite, similar to those produced by biotically generated superoxide. Yet, in contrast to the large particulate Mn oxides formed by biogenic superoxide, abiotic Mn oxides did not ripen to larger, more crystalline phases. This suggests that the deposition of crystalline Mn oxides within the environment requires a biological, or at least organic, influence. This work provides the first direct evidence that, under conditions relevant to natural waters, oxidation of Mn(II) by superoxide can occur and lead to formation of Mn oxides. For organisms that oxidize Mn(II) by producing superoxide, these findings may also point to other microbially mediated processes, in particular enzymatic hydrogen peroxide degradation and/or production of organic ligand metabolites, that allow for Mn oxide formation.

## INTRODUCTION

Manganese (Mn) oxides are among the most reactive sorbents and oxidants within the environment, where they play a central role in the cycling of nutrients, metals, and carbon (Jenne, 1968; Sunda and Kieber, 1994). Despite the overall thermodynamic favorability of Mn(II) oxidation to Mn(IV) oxides by molecular oxygen (O_2_), the first electron transfer step forming Mn(III) poses an aboitic reactivity barrier to this reaction and is subsequently the rate controlling step (Luther III, 2010). Consequently, homogeneous oxidation of aqueous Mn(II) by O_2_ is considered negligible in environments with a pH below 9 (Morgan, 2005; Luther III, 2010). The adsorption of Mn(II) to mineral surfaces (Junta and Hochella, 1994; Madden and Hochella Jr., 2005) or complexation to high-affinity ligands (Duckworth and Sposito, 2005), however, allows for the oxidation to Mn(III/IV) oxides. Further, Mn(II)-oxidizing microorganisms belonging to both the bacterial and fungal domains of life are widespread and believed to be dominant drivers of Mn oxide formation within the environment (Miyata et al., 2004; Tebo et al., 2005; Santelli et al., 2010).

In contrast to the energetically prohibitive reaction between Mn(II) and molecular oxygen, the oxidation of Mn(II) by the reactive oxygen species (ROS) superoxide ($O_{2}^{-}$) is thermodynamically favorable over all relevant pH conditions (0–13; Luther III, 2010). Indeed, the ability of Mn(II) to scavenge superoxide has been well documented (Archibald and Fridovich, 1982; Cabelli and Bielski, 1982, 1984; Nico et al., 2002; Barnese et al., 2008) and Mn(II) is touted as an important antioxidant in biological systems (Daly et al., 2004). In seawater and simulated freshwater, nanomolar levels of Mn(II) are able to scavenge superoxide, indicating a fast reaction which, at the superoxide levels previously measured in seawater (Rose et al., 2008; Hansard et al., 2010), should lead to a rapid Mn(II/III) cycle (Hansard et al., 2011). While Mn oxides could form as the result of such a cycle, Hansard et al. (2011) did not attempt to observe their formation at the low levels of Mn used in their experiments. However, in another study, formation of ROS, primarily superoxide, upon illumination of terrestrial organic carbon was implicated in the oxidation of Mn(II) to Mn oxide minerals (Nico et al., 2002). Thus, the reaction between Mn(II) and superoxide is likely important to the rates of both oxidation of Mn(II) and reduction of superoxide to hydrogen peroxide (H_2_O_2_) in aqueous environments (reaction 1).

(1) $$Mn\left(II\right)+O_{2}^{-}+{2H}^{+}\to Mn\left(III\right)+H_{2}O_{2}$$

The production of superoxide, thought to be ubiquitous in natural surface waters, has historically been attributed to photochemical reactions, yet recent research has revealed that previously unrecognized biological sources contribute, in some cases substantially, to the dark production of ROS in both terrestrial and marine systems (Kustka et al., 2005; Rose et al., 2008; Hansard et al., 2010; Saragosti et al., 2010; Vermilyea et al., 2010; Rusak et al., 2011). In fact, enzymatic extracellular superoxide production by a marine bacterium (*Roseobacter* sp. AzwK-3b) within the widespread and numerically abundant *Roseobacter* clade (Buchan et al., 2005; Hansel and Francis, 2006) has recently been found responsible for this organism’s ability to oxidize Mn(II) (Learman et al., 2011a). This biological superoxide-based Mn(II) oxidation pathway ultimately results in the precipitation of Mn oxides by way of a Mn(III) intermediate. Similarly, it was recently shown that Mn(II) oxidation by a number of common ascomycete fungi was a consequence of extracellular superoxide production during cell differentiation and reproduction (Hansel et al., 2012; Tang et al., 2013). In comparison to bacteria, fungal extracellular superoxide production has long been appreciated and shown to play important roles in fungal physiology and species interactions (Aguirre et al., 2005). However, the widespread production of extracellular superoxide by environmentally relevant heterotrophic bacteria has recently been recognized (Diaz et al., 2013). Considering superoxide’s ubiquity in the environment and recognized role in (a)biological Mn(II) oxidation, reaction between superoxide and aqueous Mn(II) may be an important pathway for Mn oxide formation within the environment.

It is unclear, however, if the formation of Mn oxides can proceed via a reaction solely between Mn(II) and superoxide or if other conditions (and/or reactants) are required for nucleation and/or precipitation to occur. For example, upon reaction of Mn(II) and superoxide (reaction 1), Mn(III) could be reduced back to Mn(II) by $O_{2}^{-}$ (reaction 2; Hansard et al., 2011), back-react with H_2_O_2_, re-forming Mn(II) but not superoxide (Archibald and Fridovich, 1982; reaction 3), be further oxidized (reaction 4) or disproportionate to Mn(II) and Mn(IV) (reaction 5). Mn(IV) is unstable as an aqueous ion and thus rapid hydrolysis will result in spontaneous precipitation of Mn(IV) as a pure or mixed valence oxide (reaction 6). Thus, only the latter two scenarios (reactions 4 and 5) of those considered would result in the formation of Mn oxide solid-phase products (reaction 6). 

(2) $$Mn\left(III\right)+O_{2}^{-}\to Mn(II)+O_{2}\,$$

(3) $$Mn(III)+1/2H_{2}O_{2}\to Mn(II)+1/2O_{2}+H^{+}$$

(4) $$Mn\left(III\right)+1/4O_{2}+H^{+}\to Mn\left(IV\right)+1/2H_{2}O\,$$

(5) 2Mn(III)→Mn(II)+Mn(IV)

(6) $$\,\;Mn\left(IV\right)+2H_{2}O\to {MnO}_{2\left(s\right)}+4H^{+}$$

Thus, despite observations of a link between Mn(II) and superoxide in Mn(II) oxidation, the potential for this reaction to directly generate solid-phase Mn(III/IV) oxides is not known. Here we address this uncertainty by conducting experiments reacting Mn(II) with abiotically generated superoxide under various conditions. We elucidate conditions necessary for the formation of Mn(III/IV) oxide minerals and also the compositional and structural properties of the ensuing Mn oxides. This work reveals a tightly coupled cycle between ROS and Mn that influences the stability of reactive intermediates and propensity for Mn oxide formation. This information will assist in identifying the geochemical environments that can support Mn oxide formation and additional microbial and potentially enzymatic processes that may be mediated by organisms oxidizing Mn(II) via superoxide. 

## MATERIALS AND METHODS

### MATERIALS

SOTS-1 [superoxide thermal source; di-(4-carboxybenzyl)hyponitrite] technical grade (Cayman Chemical) was used as a thermal source of superoxide (Ingold et al., 1997; Heller and Croot, 2010). SOTS-1 stock solutions (10 mg/mL) were made with N_2_-purged dimethyl sulfoxide (DMSO, Sigma). Solutions of artificial seawater (ASW, 0.3 M NaCl, 0.05 M MgSO_4_, 0.01 M CaCl_2_, and 0.01 M KCl; Tebo et al., 2007) contained 20 mM 4-(2-hydroxyethyl)piperazine-1-ethanesulfonic acid (HEPES; EMD) adjusted to pH 7.6. Specific enzymes used to scavenge superoxide and hydrogen peroxide were superoxide dismutase (SOD, Sigma) and catalase (Sigma), respectively. In some conditions, soluble sodium citrate (Alfa Aesar), sodium pyrophosphate (J.T. Baker), humic acid (Sigma), bovine serum albumin (BSA, Sigma), or casamino acids (Sigma) were added as a supplement to the experiment.

### Mn(II) OXIDATION EXPERIMENTS

Experiments contained 75% ASW and HEPES in deionized water with 200 μM MnCl_2_, and 1 mM SOTS-1. Experiments were conducted at room temperature and allowed to incubate for 18–24 h. Hydrogen peroxide was scavenged from experiments with the addition of 200 U of catalase to 1 mL of reaction solution at time 0, 3, and 6 h (the decomposition of catalase on a time scale of hours in our systems necessitated making several additions). Some of the experiments were supplemented with SOD (10 or 50 μM), sodium pyrophosphate (0.5 mM), sodium citrate (0.5 and 5 mM) and/or 10 mg/L humic acid. To make the particles that were analyzed spectroscopically, 1700 U of catalase were added approximately every 3 h over a period of 27 h.

### QUANTIFICATION

Oxidized Mn was quantified by monitoring the spectroscopic (Cary 60 UV–Vis spectrophotometer, Varian) absorption (620 nm) of the samples with the addition of the colorimetric dye leucoberbelin blue (LBB, Sigma; Krumbein and Altmann, 1973), which reacts with both Mn(III) and Mn(IV). Standard curves were prepared using permanganate, KMnO_4_ (EMD). To convert moles of KMnO_4_ to moles of Mn(IV) oxide or Mn(III), conversion factors of 2.5 or 5, respectively, were used, since each mole of KMnO_4_ can oxidize 2.5 or 5 times as much LBB as a mole of Mn(IV) oxide or Mn(III) (Johnson and Chiswell, 1993).

### MINERAL ANALYSIS

Mn oxides were harvested by either filtration (0.2 μm) or centrifugation (12,000 × *g* for 25 min). The collected Mn oxides were washed with distilled water and frozen at -20°C.

Transmission electron microscopy (TEM) was performed at the University of Oklahoma Samuel Roberts Noble Electron Microscopy Laboratory. Pelleted mineral samples were thawed, resuspended in ultrapure (18.2 Mømega) water, and sonicated. The liquid samples were loaded on lacey-carbon TEM grids (Ted Pella). TEM imaging was performed on a JEOL 2010-F TEM at 200 kV. Measurement and fast Fourier transform (FFT) analysis were performed with Digital Micrograph (Gatan, Inc.) and the DiffTools plugin (Mitchell, 2008).

Mn oxides were examined by X-ray absorption spectroscopy (XAS), in particular X-ray absorption near edge spectroscopy (XANES) for average oxidation state and extended X-ray absorption fine-structure (EXAFS) spectroscopy for structural information. Mn K-edge XAS spectra were collected on beamline 11-2 at the Stanford Synchrotron Radiation Lightsource (SSRL) using a Si(220) monochromator (Φ = 90°). Calibrations were made using a KMnO_4_ standard (6543.34 eV). Fluorescence data were collected with a 30-element Ge solid-state detector array with soller slits and Cr filters. Spectra were collected (three to four scans per sample) at room temperature from -200 to approximately +1000 eV around the Mn K-edge (6539 keV). Data analysis of sample spectra was performed using the SIXPACK software program (Webb, 2005). XAS scans were averaged, background-subtracted, normalized, and deglitched if necessary. The absorption edge of the Mn K-edge XANES spectra was used to estimate the proportions of Mn(II), Mn(III), and Mn(IV) by conducting linear combination fitting (LCF) with the three model Mn compounds MnCl_2_, MnOOH (feitknechtite), and δ-MnO_2_ (average oxidation state ~3.9), respectively (Villalobos et al., 2003; Bargar et al., 2005; Webb et al., 2005a). The normalized absorption spectra were analyzed using a data range of 6560–6600 eV. Binding energies were fixed and negative component contributions were prohibited for LCF. The goodness of fit was established by minimization of the *R*-factor parameter (Newville, 2001). Previous investigations have defined the 1σ error estimates to be 1.7, 2.6, and 2.9% for Mn(II), Mn(III), and Mn(IV), respectively (Bargar et al., 2005).

For EXAFS analysis, the χ(*k*) spectra were *k*^3^-weighted and analyzed using a *k* range of 3–12 Å^-^^1^. LCF was performed using model compounds as described previously (Bargar et al., 2005) and include δ-MnO_2_, hexagonal Na-birnessite, triclinic Ca-birnessite, groutite (α-MnOOH), feitknechtite (β-MnOOH), manganite (γ-MnOOH), hausmannite (Mn_3_O_4_), synthetic todorokite [(Na,Ca,K)(Mg,Mn)Mn_6_O_14_.5H_2_O], pyrolusite (β-MnO_2_), synthetic Mn_2_O_3_, aqueous Mn(III) pyrophosphate, aqueous MnCl_2_, and aqueous MnSO_4_. For LCF, binding energies were not allowed to float, a negative component contribution was prohibited, and components were not summed to 1.0.

## RESULTS AND DISCUSSION

### Mn OXIDE PRECIPITATION

**FIGURE 1:**
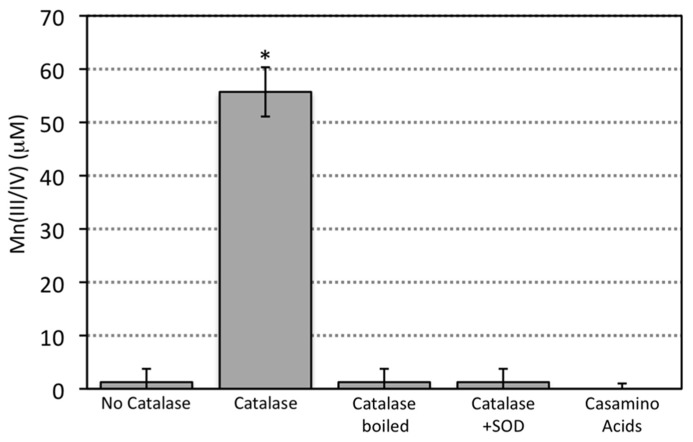
**Reaction of SOTS (1 mM) produced superoxide with Mn(II) (200 μM) in the absence or presence of catalase, boiled catalase, SOD (10 μM), and Casamino acids.** Casamino acids contain a mixture of single amino acids and small peptides obtained from the hydrolysis of casein. Measurements are presented as the sample mean ± SD for replicate samples (*n* = 3). Asterisks indicate treatments that significantly (*p* < 0.05) differ based on the Student’s *t*-test (unpaired, two-tailed) from the control condition (no catalase).

Mn oxide minerals were not observed upon reaction between abiotically generated superoxide and aqueous Mn(II) (**Figure 1**). In ASW (75%) buffered to a pH of 7.6, 200 μM aqueous Mn(II) (as MnCl_2_) was reacted at room temperature with 1 mM of the chemical superoxide source (SOTS-1; Ingold et al., 1997; Heller and Croot, 2010), which generates an initial superoxide flux of about 6 nM s^-^^1^ (Heller and Croot, 2010). Following several days of reaction, visible Mn oxide precipitates or discoloration of the ASW solution was not observed. Addition of the colorimetric dye LBB (Krumbein and Altmann, 1973), which oxidizes and turns blue in the presence of both Mn(III) and Mn(IV), showed no reaction, confirming the absence of oxidized Mn within the reacted solution (**Figure 1**). Since LBB reacts with both Mn(III) and Mn(IV), these results indicated that either (1) Mn(II) was not oxidized by superoxide or that (2) the intermediate product Mn(III) was being reduced back to Mn(II) (Archibald and Fridovich, 1982) before Mn(IV) could form and spontaneously precipitate. Considering the demonstrated ability of superoxide to oxidize Mn(II) at these and lower superoxide fluxes and Mn(II) concentrations (Archibald and Fridovich, 1982; Nico et al., 2002; Hansard et al., 2011; Learman et al., 2011a), we hypothesized that the latter explanation was responsible for the lack of Mn oxide formation.

Indeed, when catalase, a protein that catalyzes the decomposition of H_2_O_2_ to H_2_O and O_2_ (reaction 7) was added to the reaction, oxidized Mn was detected (**Figure 1**) and the ASW obtained a brown hue hinting at the presence of Mn oxide minerals (verified with LBB, see mineral discussion below and **Figures 4** and **5**).

(7) $$H_{2}O_{2}\overset{catalase}{\to }H_{2}O+1/2O_{2}\,$$

Mn oxide precipitation was negated when the catalase enzyme was boiled prior to addition, thus removing its catalytic activity (**Figure 1**). These results indicate that the enzymatic ability of the catalase to degrade H_2_O_2_ was requisite for Mn oxide formation. Mn oxide formation was not observed upon addition of other general (non-functional) proteins (e.g., BSA) or amino acids (e.g., casamino acids; **Figure 1**), ruling out the possibility of a non-specific protein or amino acid effect. Molecular oxygen is generated through the degradation of hydrogen peroxide by catalase (reaction 7) and thus it may be suggested that increased oxygen concentration could increase the propensity for Mn(II) oxidation. Yet, when SOD, an enzyme that scavenges superoxide and produces molecular oxygen and hydrogen peroxide (reaction 8), was added in the presence of catalase (a condition that would result in the highest molecular oxygen concentrations tested here), Mn oxide formation again was not observed (**Figure 1**).

(8) $${2O}_{2}^{-}+{2H}^{+}\to O_{2}+H_{2}O_{2}$$

Taken together, these results reveal that both the presence of superoxide and removal of H_2_O_2_ are required for Mn oxide formation. The product of Mn(II) reaction with superoxide, H_2_O_2_, likely inhibits the formation of Mn oxides. This inhibition is likely due to a back reaction between Mn(III) and H_2_O_2_ (reaction 3) as observed previously (Archibald and Fridovich, 1982) but could also be a result of thermodynamic inhibition by increased H_2_O_2_ levels. Either way, scavenging of H_2_O_2_ is a requisite step in the formation of Mn oxides by superoxide reaction with Mn(II).

Measuring loss of Mn(III) under these conditions is complicated by the need to complex Mn(III) to measure it spectrophotometrically (Webb et al., 2005b; Madison et al., 2011), yet these complexes will impact the kinetics of superoxide reaction with Mn(II) and the reaction progression to Mn oxides. Reactions conducted in the presence of complexing ligands (see below), however, confirm that Mn(III) is formed upon reaction of Mn(II) and superoxide as predicted and demonstrated previously (Archibald and Fridovich, 1982; Learman et al., 2011a).

We can estimate the concentration of H_2_O_2_ maintained by the catalase in our system with a simple steady-state calculation. A unit of catalase is defined as the amount capable of degrading 1 μmole of H_2_O_2_ per minute at pH 7 at 25°C in the presence of 50 μM H_2_O_2_ (Sigma-Aldrich). Since catalase has a very high half-saturation constant (~1 M; Ogura, 1955), we can assume that the rate of H_2_O_2_ degradation is first order with respect to both catalase and H_2_O_2_ concentration. From the definition of a unit, we calculate a second-order rate constant of 2 × 10^-^^5^ U L^-^^1^ min^-^^1^, neglecting the slight difference between our reaction temperature at 25°C. The concentration of catalase added to our systems at *t* = 0 was 2 × 10^5^ U L^-^^1^, giving a pseudo-first order decay rate coefficient of 4 min^-^^1^. Equating the initial rate of H_2_O_2_ production (estimated as equal to the rate of superoxide production from 1 mM SOTS at room temperature; Heller and Croot, 2010) with its rate of decay, we obtain

$$360\,\;nM\,\;\min^{-1}=4\,\;\min^{-1}\left[H_{2}O_{2}\right]$$

Thus, the catalase in this system will keep H_2_O_2_ levels below ~90 nM, which allows the precipitation of Mn oxides. In contrast, without catalase, H_2_O_2_ would be expected to build up to a concentration exceeding several micromolar within 10 min.

### Mn OXIDE FORMATION IN THE PRESENCE OF COMPLEXING LIGANDS

**FIGURE 2:**
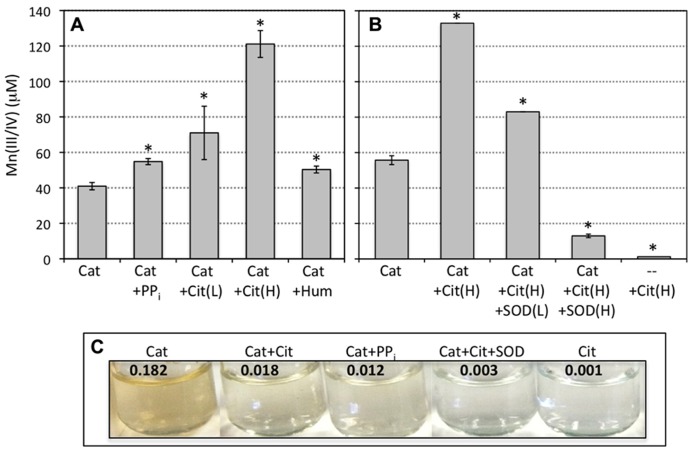
**(A)** Abiotically produced superoxide (1 mM SOTS) reacted with Mn(II) (200 μM) in the absence and presence of catalase (Cat) and pyrophosphate (0.5 mM; PP_i_), citrate (0.5 and 5 mM, Cit(L) and Cit(H), respectively) or humic acids (10 mg/L). **(B)** Abiotically produced superoxide reacted with Mn(II) in the presence of 5 mM citrate and catalase and/or SOD (10 and 50 μM). Measurements are presented as the sample mean ± SD for replicate samples (*n* = 3). Asterisks indicate treatments that significantly (*p* < 0.05) differ based on the Student’s *t*-test (unpaired, two-tailed) from the control condition (catalase only). Brackets for SDs below 3% of the mean value are smaller than the width of the line. Intra-experiment variability was low (as evidenced by the low SDs) but there was slight inter-experiment variability due to minor deviations in reaction time, temperature in the lab, and SOTS concentration. Individual experiments are plotted separately for direct comparison to the corresponding control condition for each experiment. **(C)** Pictures depict the color of a subset of the reacted solutions following 24 h of reaction. The numbers at the top of pictures indicate the visible light absorbance at 700 nm, which is indicative of the Mn oxide colloid contribution. Background absorbance for SOTS reaction with 200 μM Mn(II) in the absence of catalase is 0.001.

In light of the results described above, the stability and/or preservation of Mn(III) has a direct bearing on the formation of Mn oxide minerals generated from reactions between aqueous Mn(II) and superoxide. Thus, the presence of organic or inorganic ligands that complex Mn(III) may result in enhanced stabilization of Mn(III) to resist back reaction with H_2_O_2_ and allow for Mn oxide formation. Citrate and pyrophosphate have the demonstrated abilities to complex and stabilize aqueous Mn(III) (Klewicki and Morgan, 1998; Webb et al., 2005b). Indeed, the addition of pyrophosphate (0.5 mM) and citrate (0.5 and 5.0 mM) to the SOTS-Mn(II)-catalase reaction significantly (*p* < 0.05) increased the LBB signal (**Figure 2A**). Once again, Mn(III/IV) was not detected in the absence of catalase. These results suggest that pyrophosphate and citrate played a role in stabilizing Mn(III), but did not eliminate the inhibition of net Mn(III) formation by high H_2_O_2_. Further, addition of humic acids also increased the formation of oxidized Mn relative to the control (catalase only; **Figure 2A**). All reactions were conducted in the dark, eliminating light-induced electron transfer reactions.

In addition, complexation by citrate appeared to accelerate the reaction of Mn(II) with superoxide. 10 μM of the superoxide scavenger SOD, sufficient to eliminate Mn oxidation in the absence of ligands (**Figure 1**), decreased the amount of Mn(II) oxidized in the presence of citrate and catalase by only ca. 40% relative to that in the absence of SOD, while 50 μM decreased Mn(II) oxidation by more than 90% (**Figure 2B**). No oxidation was observed in the absence of SOTS (the superoxide source). This indicates that superoxide was still the sole oxidant of Mn(II), but that the reaction of Mn(II)-citrate with superoxide is faster than the reaction of uncomplexed Mn(II), requiring more SOD to outcompete Mn for the superoxide. This also indicates that oxidation of Mn(II)-citrate complexes by molecular oxygen or direct electron transfer as observed previously (Klewicki and Morgan, 1998) was not important on the time scale of our experiments. Thus similar to conditions in the absence of citrate, both the presence of superoxide and elimination of hydrogen peroxide are required for net Mn(II) oxidation under these experimental conditions.

**FIGURE 3:**
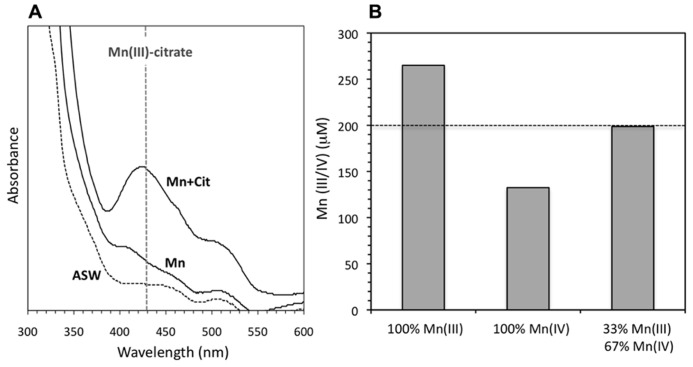
**(A)** UV-visible absorbance scan (300–600 nm) for solutions containing solely ASW + catalase (ASW), ASW + Mn(II) + catalase (Mn), and ASW + Mn(II) + catalase + 5 mM citrate (Mn + cit). All Mn(II) concentrations are 200 μM and all solutions contain SOTS. Vertical dotted line illustrates the absorbance wavelength for Mn(III)-citrate complexes. Solutions were centrifuged before scanning to remove the contribution of Mn oxide colloids to the absorbance. **(B)** Oxidized Mn concentrations calculated from the LBB measurement. If we assume oxidized Mn is solely Mn(III) (left-most bar), the total oxidized Mn is higher than the initial Mn(II) present in the system. A mixture of Mn(III) and Mn(IV) gives a total concentration of oxidized Mn in between these extremes. The rightmost bar illustrates that a mixture of 33% Mn(III) and 67% Mn(IV) corresponds to oxidation of all the initial Mn(II)).

Yet, although more oxidized Mn was observed in the presence of organic ligands, a lower proportion was present as a solid-phase, based both on the appearance of the solutions and on absorbance measurements. The reacted solutions in the absence of (in)organic ligands were darker (more brown) than equivalent conditions in the presence of those same ligands (**Figure 2C**), implying lower concentrations of Mn oxide colloids in the latter case (see discussion of solid-phases below). Under non-Mn oxide forming conditions, that is, in the absence of catalase, background absorbance at 700 nm for SOTS reaction with Mn(II) ranged from 0.001 to 0.004, including conditions containing citrate, pyrophosphate, and humic acids (**Figure 2C**). In contrast, the absorbance signal (caused mostly by light scattering) from colloidal Mn oxides formed upon reaction of SOTS and Mn(II) in the presence of catalase was 0.182 but was an order of magnitude lower in the presence of citrate (0.018), pyrophosphate (0.012) and humic acids (0.016), again indicating lower Mn oxide content. Furthermore, when solutions containing both catalase and citrate were centrifuged to decrease the contribution of colloidal Mn to the absorbance signal, a strong absorbance peak at 430 nm indicative of a Mn(III)-citrate complex (Klewicki and Morgan, 1998) appeared (**Figure 3A**). As to be expected, this complex was only observed in the presence of citrate.

However, all oxidized Mn could not have been present as Mn(III) as in that case the oxidized Mn measured by LBB would exceed (~260 μM) the total Mn added (200 μM) to the reaction containing 5 mM citrate (**Figure 3B**). We can calculate the maximum possible amount of Mn(III) that could have formed by assuming that all of the Mn(II) oxidized either to Mn(III) or to Mn(IV); in this case, final concentrations of 65 μM (33%) of Mn(III) and 135 μM (67%) Mn(IV) would be more consistent with the measured LBB signal in the presence of 5 μM citrate (**Figure 2**). Together, this evidence suggests that in the presence of citrate, both Mn(III)-citrate complexes and Mn oxide colloids are present. This is consistent with the results of Klewicki and Morgan (1998), who found that Mn(III)-citrate complexes could disproportionate to form Mn oxide precipitates.

Citrate has redox active properties and can induce Mn oxide reductive dissolution (Wang and Stone, 2006). This reaction can lead to redox cycling of Mn at the Mn oxide surface producing Mn(II) and ultimately Mn(III)-citrate. Thus, following the initiation of Mn oxide precipitation, reactions between citrate and the Mn oxide surface may also contribute to the preferential accumulation of Mn(III)-citrate complexes over Mn oxide particles observed here (**Figures 2** and **3**). This contribution would increase as the concentration of citrate increases (Wang and Stone, 2006). However, this mechanism cannot be the sole source of Mn(III)-citrate in our systems, since it would not explain our observations that much more Mn is oxidized in the presence of citrate than in its absence, and that the effectiveness of SOD in quenching Mn oxide formation is decreased in the presence of citrate. In addition, higher concentrations of oxidized Mn yet lower Mn oxide precipitation were also observed in the presence of pyrophosphate (**Figure 2**) which does not have these redox-active properties, further supporting a role for Mn(III) complexation in the net accumulation of oxidized Mn in the presence of ROS.

### CHARACTERISTICS OF SUPEROXIDE-GENERATED Mn OXIDES

**FIGURE 4:**
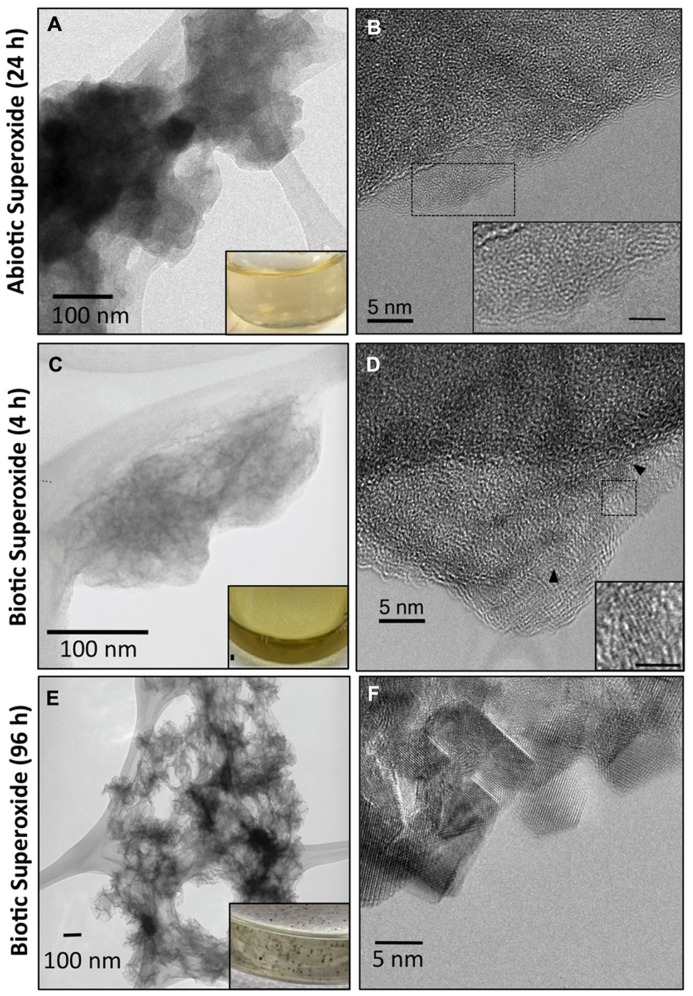
**Transmission electron microscopy (TEM) images (A,C) illustrating small dispersed, colloidal Mn oxides formed via oxidation of Mn(II) by abiotically **(A)** and biotically generated **(C)** superoxide and 24 and 4 h, respectively.** TEM images of Mn oxides formed by biotic superoxide after 96 h show mineral particle aggregates **(E)**. Texture underneath the particles is due to the lacey-carbon support film. Insets in **(A,C,E)** are pictures of the reacted solution appearance. Biotic superoxide Mn oxides were formed via reaction of superoxide-generating microbial cell-free filtrate with Mn(II) as described in detail previously (images modified from Learman et al., 2011b). High resolution TEM **(B,D,F)** demonstrating poor crystallinity of the superoxide-produced Mn oxides with a lack of discernable lattice fringes in abiotic generated Mn oxides **(B)** and small (~5 nm) crystalline domains in biotic Mn oxides **(D)**. HR-TEM images of Mn oxides formed by biotic superoxide after 96 h, however, show defined crystalline domains **(F)**. Insets in **(B,D)** illustrate the difference in crystallinity (scale bar = 2 nm).

In the presence of catalase, reactions between Mn(II) and superoxide led to a discoloration of the ASW from clear to light brown (**Figure 2C**), indicative of Mn oxide formation as quantified by LBB (see **Figure 1**). These Mn oxides were harvested (24 h of reaction) by centrifugation (see Materials and Methods) resulting in a thin brown film on the centrifuge tube wall. TEM revealed that the brown film was composed of small, dispersed colloidal Mn oxides averaging 100–125 nm in diameter with no evidence of aggregation (**Figure 4A**). The interiors of the oxide grains appeared highly disordered and discernable lattice fringes were not observed via high resolution TEM (HR-TEM) imaging, indicating that the oxides were very poorly crystalline, if not “amorphous” (**Figure 4B**). The lack of lattice fringes suggested the particles were strained, perhaps attributable in part to a poorly ordered distribution of interlayer cations. These poorly crystalline colloidal Mn oxides were stable under these conditions, and ripening or aggregation-based growth was not observed following several days at room temperature (see below).

**FIGURE 5:**
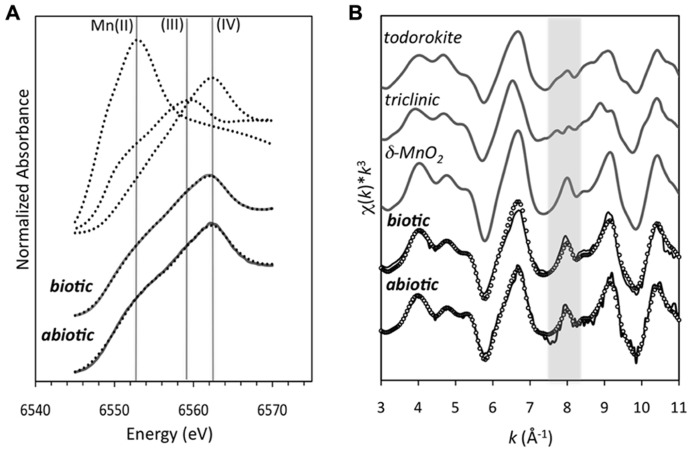
**(A)** Mn K-edge XANES spectra of the Mn oxides produced from biotically produced Mn oxides after 4 h (after Learman et al., 2011b) and abiotically produced Mn oxides. The dotted lines represent the standards for Mn(IV) (δ-MnO_2_), Mn(III) (feitknechtite, β-MnOOH), and Mn(II) (MnCl_2_; as described in Bargar et al., 2005). The solid gray line is the linear combination fit (LCF) using the three Mn standards to represent the three oxidation states. **(B)** *k*^3^-weighted Mn EXAFS spectra (solid line) and linear-combination fit (dotted line) for the biotically and abiotically produced Mn oxides. The gray shaded area highlights the “indicator region” emphasizing the spectral differences that can be used to distinguish hexagonal (as δ-MnO2) from both triclinic birnessite (labeled triclinic) and todorokite (as described in Webb et al., 2005a).

The Mn oxides were composed of a poorly ordered, poorly crystalline phyllomanganate with hexagonal symmetry and low Mn(III) content, similar to δ-MnO_2_ (**Figure 5**). The energy position of the XANES absorbance maximum for the abiotic Mn oxide centers around 6562 eV. This position is consistent with the absorbance maximum of δ-MnO_2_, which has an average oxidation state of 3.9–4.0, suggesting that the oxides are dominated by Mn(IV). LCF of the XANES spectra using δ-MnO_2_, feitknechtite (β-MnOOH), and MnCl_2_ indicated approximate relative percentages of Mn(IV), Mn(III), and Mn(II) as 82, 5, and 13%, respectively (**Figure 5**; *R*-factor = 0.07) – yet, due to the difficulty in assigning a specific binding energy to Mn(III), the values obtained should be taken as approximate values. The Mn K-edge EXAFS spectrum of the abiotic Mn oxides could be fully reconstructed with solely δ-MnO_2_ by LCF (**Figure 5**; *R*-factor = 0.05). Indeed, visual examination of the Mn K-edge spectral fingerprints for model compounds of the three most common biogenic oxides (**Figure 5**) illustrate differences in the features at 6.8, 8.0 (the “indicator” region), and 9.3 Å^-^^1^, which are diagnostic of phyllomanganates (McKeown and Post, 2001). In particular, while hexagonal birnessite (as δ-MnO_2_ in **Figure 5**) has a distinct sharp peak at ~8 Å^-^^1^, a decrease in the amplitude of this oscillation and a broadening of the feature at ~9 Å^-^^1^ is consistent with an increase in the triclinic birnessite and/or todorokite content (McKeown and Post, 2001; Gaillot et al., 2003; Manceau et al., 2004; Webb et al., 2005a). Here, the EXAFS spectrum for the abiotic Mn oxide has a shoulder at 6.8 Å^-^^1^, one sharp peak at 8.0 Å^-^^1^, and a sharp peak at 9.3 Å^-^^1^, all characteristic of hexagonal birnessite (Gaillot et al., 2003; Manceau et al., 2004).

Interestingly, the abiotic Mn oxides observed here are structurally similar to those produced biotically by a large taxonomically diverse group of organisms (fungi and bacteria) that likely employ different enzymatic and oxidation pathways (Jurgensen et al., 2004; Bargar et al., 2005; Webb et al., 2005a; Grangeon et al., 2010). In fact, the Mn oxides formed here in a purely abiotic superoxide system were similar to those previously characterized upon reaction of Mn(II) with superoxide-generating microbial exudate (cell-free filtrate). The biotic oxides were formed by reacting Mn(II) for 4 h with the superoxide-generating cell-free filtrate produced by *Roseobacter* AzwK-3b grown in an organic carbon replete medium (K media, pH 7.6; Learman et al., 2011b). Similar to the abiotic oxides, the Mn K-edge EXAFS spectra of the biogenic Mn oxides were fully reconstructed with solely δ-MnO_2_ (**Figure 5**; Learman et al., 2011b). In comparison to the abiotic Mn oxides, however, the oxides formed by superoxide generated microbial filtrate contained a higher relative proportion of Mn(III), with approximate values being 8% Mn(II), 21% Mn(III), and 71% Mn(IV) (Learman et al., 2011b). In addition, the biotically generated superoxide had discrete crystalline domains – albeit small with lattice fringes continuous only over regions less than 5 nm (**Figure 4D**; Learman et al., 2011b). The coherent scattering domains of the biogenic oxides are rotated in a nearly continuous distribution of orientations indicating a high degree of disorder (**Figure 4D**), only slightly more ordered than the abiotic analogs. These crystallinity differences cannot be attributed simply to aging since the biogenic Mn oxides were in fact younger (4 h) than the abiotically generated Mn oxides (24 h).

Despite the similarity of initial Mn oxides, the geochemical conditions within which the Mn(II) and superoxide react influences ripening and aggregation of the oxides to more crystalline, ordered phases. After several days of reaction between Mn(II) and abiotically generated superoxide, the cloudy brown color indicative of the colloidal Mn oxides remained unchanged and visible Mn oxide particles and/or aggregates were not observed. In contrast, when Mn(II) reacted with biogenic superoxide within cell-free microbial filtrate, the brown hue disappeared after 96 h and Mn oxide minerals were clearly visible by eye, with particle size continuing to grow over time (**Figure 4**; Learman et al., 2011b). Indeed, well-defined lattice fringes were apparent in nearly every region of the grain, although micron-sized grains were aggregates of crystallites ranging from five to tens of nanometers in diameter. In light of the similar initial Mn oxide products, the ability of the superoxide generated Mn oxide colloids to undergo aggregated crystal growth apparently requires another reactant that is present within the microbial exudate and may include individual or complex proteins, organic metabolites, or extracellular organic polymers. This organic-mediated aggregation could lead to the large morphological diversity of structurally similar Mn oxides formed under various biological conditions (e.g., fungi, bacteria, cell-free filtrates; Emerson et al., 1989; van Waasbergen et al., 1996; Villalobos et al., 2003; Tebo et al., 2004; Toner et al., 2005; Miyata et al., 2006a; Learman et al., 2011b; Santelli et al., 2011; Tang et al., 2013). Further, the mineral associated organics may lead to the observed higher proportion of Mn(III) within the biogenic oxides compared to the abiotically generated oxides.

## CONCLUSION

This work provides the first direct evidence that, under conditions relevant to natural waters, oxidation of Mn(II) by superoxide can occur and lead to formation of Mn oxides. These results are consistent with previous observations of Mn oxide formation during photo-oxidation of humic substances (Nico et al., 2002), where we suspect the humic material also served to stabilize Mn(III) to allow for oxide precipitation. Our data also indicate that H_2_O_2_ inhibits Mn oxidation, possibly because the intermediate Mn oxidation product, Mn(III), rapidly oxidizes H_2_O_2_, regenerating Mn(II) (e.g., reaction 3). Finally, we have demonstrated here that the presence of (in)organic ligands can increase the yield of oxidized Mn but decreases net oxide formation, likely by stabilizing Mn(III). Recent studies have highlighted the ubiquity and abundance of Mn(III)-ligand complexes in aqueous and sedimentary environments (Trouwborst et al., 2006; Madison et al., 2011). The reaction of Mn(II) with superoxide is one possible source of these complexes and their formation and stability will ultimately impact the precipitation of Mn.

Could reaction between superoxide and Mn(II) represent a source of Mn oxide minerals in environmental systems? Our results indicate that one question to consider is whether H_2_O_2_ levels remain sufficiently low for oxide formation in the presence of superoxide. The highest production rates of both superoxide and H_2_O_2_ are expected in sunlit-irradiated surface waters rich in natural organic matter. Even in such waters, H_2_O_2_ concentrations generally remain smaller than 1 μM (Scully et al., 1995; Richard et al., 2007), close to the conditions created in our experimental systems by adding catalase. Thus, while both Mn(II) concentrations and superoxide fluxes created by ultraviolet (UV)-oxidation of natural organic matter are lower than those in our experimental systems, it seems plausible that Mn oxide formation by superoxide could occur in sunlit natural waters. However, since photoreduction of Mn oxides is also known to occur (Sunda and Huntsman, 1994), the net effect of light is not necessarily Mn oxidation.

In the absence of UV light such as in deep waters and soils, both superoxide production and H_2_O_2_ production and decay will be controlled by biological activity (Rose et al., 2008; Hansard et al., 2010; Vermilyea et al., 2010) and reactions with metals, such as Fe (Rose and Waite, 2002; Burns et al., 2010). Our results indicate that as long as these processes result in both superoxide production and net H_2_O_2_ decay, Mn oxides could precipitate. Indeed, pure isolates of common marine bacteria (Learman et al., 2011a) and both marine and soil fungi (Hansel et al., 2012; Tang et al., 2013) have been found which meet these minimum requirements and are capable of oxidative precipitation of Mn via the formation of extracellular superoxide. The results provided here may point to other microbially mediated processes occurring, however, to allow for Mn oxide formation, in particular enzymatic hydrogen peroxide degradation and/or production of organic metabolites and/or polymers that can function as Mn complexing ligands.

In addition to the factors discussed above, the importance of superoxide-based Mn oxide formation in environmental systems will depend on the rate constant of the initial reaction (reaction 1) and on the concentration of superoxide present. Hansard et al. (2011) have shown that under seawater conditions, the reaction is sufficiently fast, and superoxide sufficiently abundant, to oxidize Mn(II) on a time scale on the order of hours. The same study found a similarly fast reaction rate for simulated freshwater conditions, but a time scale for Mn(II) oxidation for these conditions could not be calculated since superoxide concentrations in freshwaters are unknown. Superoxide has also not yet been quantified in soils and sedimentary environments, but could be formed both by biological activity and by redox reactions such as the oxidation of Fe(II) (Rose and Waite, 2002; Burns et al., 2010).

Another factor affecting the importance of superoxide to Mn oxide formation is the extent to which the Mn(III) produced by reaction 1 is re-reduced instead of being further oxidized. Hansard et al. (2011) observed evidence for a Mn(II/III) cycle in their seawater experiments, possibly because of reduction of Mn(III) by superoxide. Some organic compounds may also be able to react with the oxidized Mn intermediates, regenerating Mn(II) and thus preventing Mn oxide formation. Even if no Mn oxide accumulates, the rapid formation and turnover of reactive intermediate Mn species may have vast implications for the redox cycling of other (in)organic compounds, somewhat analogous to the recently revealed “cryptic” sulfur cycle (Canfield et al., 2010). Further, this rapidly spinning Mn cycle could have a substantial influence on the concentrations of superoxide and hydrogen peroxide in surface waters.

A final finding of this study was that the ensuing poorly ordered, nanocrystalline birnessite products formed upon superoxide-mediated Mn(II) oxidation are similar to Mn oxides formed by various organisms, including bacteria and fungi, that employ different mechanisms of Mn(II) oxidation (Villalobos et al., 2003; Tebo et al., 2004, 2005; Bargar et al., 2005; Webb et al., 2005a, b; Miyata et al., 2006a, b; Santelli et al., 2011). Further, the similarity in Mn oxide structure here implies that Mn oxide products may not carry an obvious signature of the process responsible for superoxide generation (e.g., UV-versus microbially generated superoxide). Nevertheless, it is important to note that despite the fact that the abiotic Mn oxides did not aggregate to larger particulate Mn oxides over time (i.e., the oxides remained colloidal), Mn oxides formed by biotically generated superoxide within live cell incubations and a cell-free filtrate rapidly formed particulate (visible) Mn oxides. Thus, despite the non-specificity of the initial Mn oxide products to reaction mechanism, observations of particulate Mn oxide minerals and deposits within the environment may point to a biological (or at least organic) influence.

## Conflict of Interest Statement

The authors declare that the research was conducted in the absence of any commercial or financial relationships that could be construed as a potential conflict of interest.
